# Thyroid-Stimulating Hormone (TSH)-Secreting Pituitary Tumor Misdiagnosed for 20 Years: Possible Effect of Long-Term Treatment With Thyroid Hormone

**DOI:** 10.7759/cureus.107985

**Published:** 2026-04-29

**Authors:** Rodhan Khthir

**Affiliations:** 1 Endocrinology, Diabetes and Metabolism, Sanford Health, University of North Dakota, Bismarck, USA

**Keywords:** central hyperthyroidism, pit-1, pituitary adenoma, tshoma, tumor biology

## Abstract

Thyroid-stimulating hormone (TSH)-secreting pituitary adenomas (TSHomas) are rare causes of central hyperthyroidism. Misdiagnosis may lead to prolonged inappropriate thyroid hormone therapy.

I present a 65-year-old woman who was treated with high-dose levothyroxine for 20 years for presumed primary hypothyroidism despite persistently elevated TSH with normal to high free T4 and T3 levels. Central hyperthyroidism was not considered. A 9-11 mm pituitary lesion remained stable for two decades. Biochemical evaluation revealed an elevated alpha-subunit with negative heterophile antibodies, confirming a TSHoma. She underwent transsphenoidal resection; pathology confirmed a plurihormonal PIT-1-positive adenoma.

This case challenges the traditional view that TSHomas are uniformly resistant to thyroid hormone feedback. The tumor’s long-term radiographic stability during supraphysiologic levothyroxine therapy raises the possibility of partial feedback sensitivity. The absence of measurable growth over two decades could be supportive of this hypothesis.

Discordant thyroid function tests should prompt evaluation for central hyperthyroidism. Biological heterogeneity among TSHomas may influence tumor behavior and therapeutic responsiveness.

## Introduction

Thyrotroph pituitary neuroendocrine tumors (PitNETs) are rare thyroid-stimulating hormone (TSH)-secreting pituitary tumors, representing less than 1% of all pituitary adenomas and constituting an uncommon cause of hyperthyroidism [1]. They are characterized biochemically by elevated circulating thyroid hormone levels with an inappropriately normal or elevated TSH, a pattern that distinguishes them from primary thyroid disorders [1-3]. Clinically, patients often present with symptoms of hyperthyroidism, though these manifestations may be less pronounced than in primary hyperthyroidism [3]. In addition, mass-effect symptoms-including headache and visual disturbances-may occur when the tumor presents as a macroadenoma [3].

The differential diagnosis of elevated thyroid hormones with non-suppressed TSH includes thyroid hormone resistance (RTHβ), an autosomal dominant condition in which patients may appear euthyroid or exhibit variable degrees of hypo- or hyperthyroid symptoms, often accompanied by neurocognitive or behavioral features and a positive family history [1-3]. Another important consideration is assay interference, particularly from heterophile antibodies, which may produce discordant thyroid function tests in otherwise euthyroid individuals [1-3].

Failure to recognize TSH-PitNETs can lead to prolonged misdiagnosis and inappropriate therapy, especially in primary care settings where reflex TSH-based testing algorithms may obscure the diagnosis [1,2]. Because the estimated incidence is only 0.3-1 per million, the natural history of these tumors remains incompletely understood, and diagnostic delays are not uncommon [1]. Some patients are mistakenly treated with antithyroid medications or supraphysiologic doses of levothyroxine, which may further complicate the clinical picture and postpone definitive evaluation [3].

This case is presented to increase awareness of TSH-PitNETs and highlight the diagnostic challenges encountered in primary care. Additionally, we describe the unusual observation that in this patient, the TSH-secreting pituitary tumor remained radiographically stable for more than 20 years on continuous supraphysiologic levothyroxine therapy, raising important considerations regarding tumor biology and potential partial feedback sensitivity.

## Case presentation

A 65-year-old woman was referred in November 2021 for evaluation of difficult-to-control hypothyroidism. She had been receiving levothyroxine therapy for nearly 20 years prior to her initial visit to the endocrine clinic and had been taking levothyroxine 300 mcg daily for an unknown number of those years. Her medical history included hypertension, atrial fibrillation, and well-controlled type 2 diabetes mellitus.

Approximately 20 years before presentation, she was diagnosed with abnormal thyroid function and was initially treated for presumed hyperthyroidism before being transitioned to long-term levothyroxine therapy. She reported strict adherence throughout this period.

**Figure 1:**
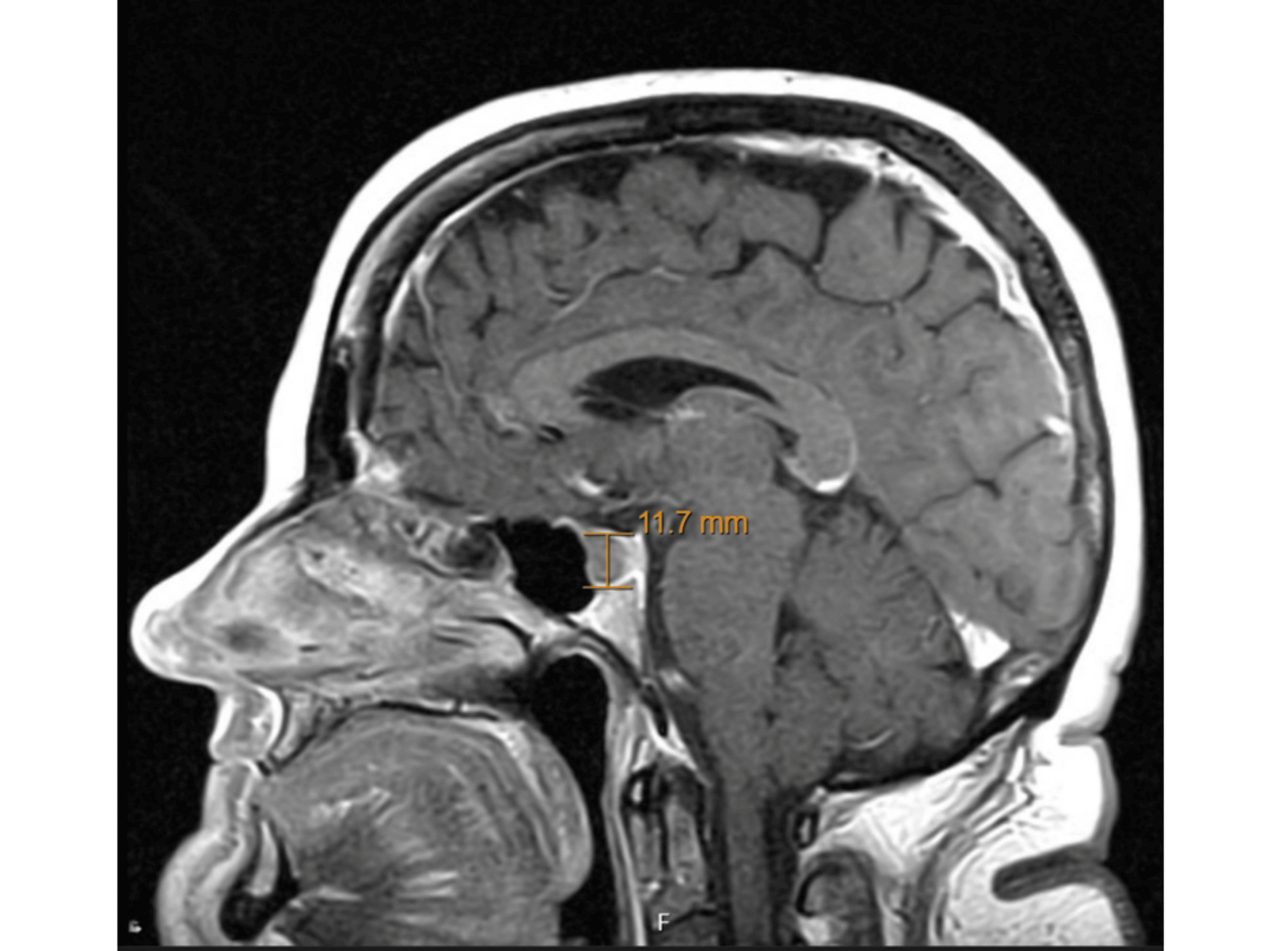
Sagittal MRI obtained four years before the initial endocrine clinic visit, demonstrating an 11.7 mm hypoenhancing lesion within the adenohypophysis with slight suprasellar extension and no evidence of mass effect.

A 10 mm pituitary lesion had been identified at that time following the discovery of an elevated prolactin level. She was treated with bromocriptine for less than two years after the initial pituitary MRI, after which her prolactin level normalized and remained normal without further therapy. The pituitary lesion was considered radiographically stable on subsequent imaging. An MRI performed four years prior to her first endocrine clinic visit demonstrated an 11.7 mm hypoenhancing lesion in the adenohypophysis with slight suprasellar extension and no optic chiasm compression (Figure 1).

During her initial evaluation at the endocrine clinic in 2021, laboratory studies revealed TSH levels fluctuating between 4 and 17 mIU/mL in the setting of normal to high free T4, raising concern for central hyperthyroidism. The combination of elevated thyroid hormones with a non-suppressed TSH prompted a differential diagnosis that included TSH-secreting pituitary adenoma (TSHoma), thyroid hormone resistance, and assay interference.

She denied headaches, visual changes, or other symptoms suggestive of mass effect. Clinically, she exhibited features consistent with hyperthyroidism, including difficult-to-control atrial fibrillation and anxiety. She had no history of learning disabilities, cognitive impairment, or hearing loss, and no family history of thyroid hormone resistance. Physical examination demonstrated irritability, mild tachycardia, elevated blood pressure, and a fine tremor. The thyroid gland was neither enlarged nor nodular.

Levothyroxine was discontinued at the initial visit. Follow-up laboratory testing showed persistent elevation of free T4 and free T3, accompanied by an elevated alpha-subunit, supporting autonomous TSH secretion. Testing for human anti-mouse antibodies (HAMA) was negative, reducing the likelihood of assay interference. Pituitary function testing demonstrated normal prolactin, normal insulin-like growth factor-1 (IGF-1), normal adrenal function, and appropriately elevated follicle-stimulating hormone (FSH) in the menopausal range.

**Figure 2:**
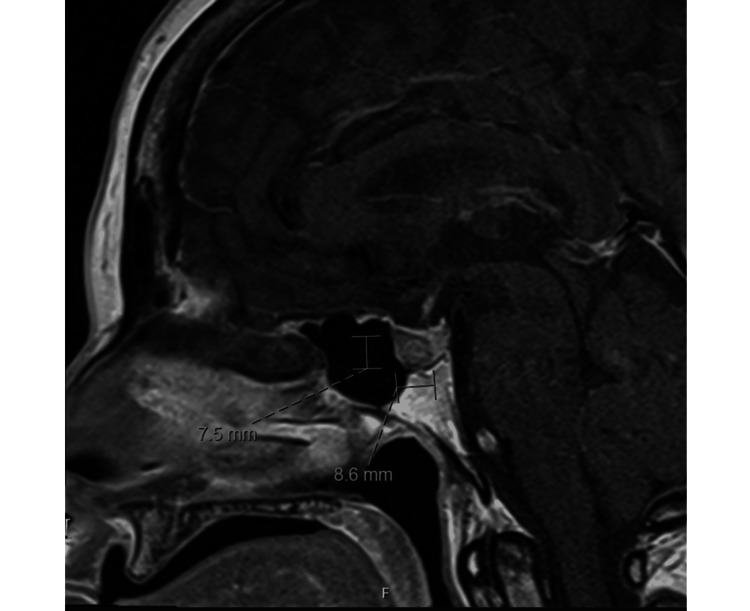
Sagittal MRI three months after initial visit and shortly before surgery, showing a 7.5 × 8.6 mm hypoenhancing lesion in the anterior sella. No suprasellar extension or optic chiasm compression.

A repeat pituitary MRI obtained three months after the initial visit showed a mild decrease in tumor size, now measuring 7.5 × 8.6 mm, with no cavernous sinus invasion (Figure 2).

Five weeks after the initial visit, octreotide therapy was initiated and titrated to 100 mcg three times daily, resulting in clear biochemical improvement. Thyroid function normalized prior to surgery, and no additional endocrine deficits emerged during medical therapy.

Three months after her initial evaluation, she underwent endoscopic endonasal transsphenoidal resection of the pituitary adenoma. Her immediate postoperative course was uncomplicated. She was discharged home on postoperative day four without requiring arginine vasopressin replacement, without glucocorticoid supplementation, and without the need for thyroid hormone therapy.

Histopathologic examination, performed at a referral center, demonstrated a monomorphic adenoma composed of cells with eosinophilic cytoplasm. Immunohistochemistry showed diffuse positivity for chromogranin, PIT-1, and alpha-subunit; focal positivity for TSH and prolactin; and negative staining for SF-1, T-PIT, GH, ACTH, FSH, LH, TTF-1, and S100. These findings were consistent with a PIT-1-lineage TSHoma.

Postoperatively, thyroid function normalized (Table 1). She maintained normal pituitary function and demonstrated no evidence of tumor recurrence on serial biochemical evaluation and follow-up MRI more than three years after surgery. Her cardiac symptoms improved substantially following tumor resection. She did not require any additional therapy, including radiotherapy or ongoing somatostatin analogue treatment. 

**Table 1:** Longitudinal hormone profile with highlighted abnormal values. Abnormal values are highlighted in bold text. TSH: thyroid-stimulating hormone

Date	TSH (0.3-4.94)	Free T4 (0.7-1.5)	Free T3 (1.7-3.7)	Alpha-subunit (<1.8)	Prolactin (3.5-20)	Comments
+2 years 6 months 25 days	3.9	0.7	-	-		
+1 year 9 months 21 days	3.55	0.9	-	-		
+9 months 1 day	3.9	0.9	2.4	1.7		6 months post-transsphenoidal surgery
+3 months 14 days	2.98	0.7	-	-		12 days post-transsphenoidal surgery
+3 months 2 days						Transsphenoidal surgery. Octreotide discontinued
+2 months 8 days	3.96	1.6	-	-		
+1 month 29 days	6.61	1.3	3.3	-		
+1 month 6 days	5.39	1.7	4.4	3.8		Octreotide started/titrated
+27 days	10.73	1.8	5.4	-		
11/2021 initial visit	7.14	2.7	4.2	4.0	11.8	First visit. Levothyroxine stopped
-4 months 29 days	2.38	1.4	-	-		
-1 year 8 months 27 days	1.66	-	-	-		
-2 years 2 days	2.23	2.0	-	-		
-3 years 11 months 2 days	6.23	2.5	-	-		
-4 years 10 months 21 days	6.33	1.9	-	-		
-19 years 1 month 2 days	7.32	-	-	-	5.9	On bromocriptine 7.5 mg daily

## Discussion

We present a patient with a thyrotroph PitNET who exhibited a highly unusual clinical course. She was treated for nearly two decades with high-dose levothyroxine for a presumed diagnosis of primary hypothyroidism before inappropriate TSH secretion was recognized. This prolonged period of supraphysiologic thyroid hormone exposure provided a unique opportunity to observe the effect of long-term levothyroxine therapy on a TSH-secreting tumor. Remarkably, her pituitary adenoma remained radiographically stable-and even demonstrated slight interval reduction in size-throughout approximately 20 years of treatment. Such long-term stability is striking given the typical behavior of these tumors.

Thyrotroph PitNETs commonly present as macroadenomas with invasive features. Approximately 80% are macroadenomas with an average diameter of 20 mm, and nearly half extend into the sphenoid sinus or suprasellar region [1,3]. In contrast, our patient’s tumor remained confined to the sella without extrasellar invasion, and surgical resection was straightforward. This clinical trajectory differs from the expected natural history of these rare tumors [3].

Thyrotroph PitNETs are traditionally characterized as autonomous tumors resistant to thyroid hormone-mediated negative feedback due to reduced or dysfunctional thyroid hormone receptor β (TRβ) [3-5]. Under normal physiological conditions, TSH synthesis and secretion are tightly regulated by hypothalamic thyrotropin-releasing hormone (TRH) and by negative feedback from circulating thyroid hormones acting through TRβ. In thyrotroph PitNETs, this regulatory network is disrupted, leading to inappropriate TSH secretion despite elevated T3 and T4 levels and sustained tumor activity [3-5]. Altered TRβ expression or function is considered the most likely mechanism underlying this impaired feedback. Additional proposed mechanisms include defective recruitment of transcriptional corepressors and abnormalities in chromatin-remodeling complexes involved in TSHβ gene repression [3].

In this patient, prolonged exposure to high-dose levothyroxine may have contributed to the tumor’s long-term stability, lack of extrasellar extension, and ease of resectability. Variable expression of thyroid hormone receptors or differences in intracellular signaling pathways may influence tumor growth kinetics and responsiveness to circulating thyroid hormones [4,6-8]. Although thyrotroph PitNETs are generally considered resistant to negative feedback, partial or heterogeneous sensitivity may exist in a subset of tumors, potentially explaining this patient’s atypical course.

This case also highlights the diagnostic challenges associated with thyrotroph PitNETs. These tumors account for <1% of all pituitary adenomas and typically present with elevated thyroid hormones and a non-suppressed or high TSH-a biochemical pattern that is easily overlooked or misinterpreted [1-3]. Several factors contribute to diagnostic delay, including their rarity, limited clinician familiarity with central hyperthyroidism, and overlap with conditions such as RTHβ or assay interference. Some patients are treated with thyroid hormone replacement or antithyroid medications before inappropriate TSH secretion is recognized. In our patient, the diagnosis was delayed for more than 20 years, during which she received escalating doses of levothyroxine for a non-suppressible TSH without consideration of alternative etiologies. Greater awareness of the clinical, biochemical, and pathophysiological features of thyrotroph PitNETs is essential to avoid delayed diagnosis and prevent unnecessary or inappropriate treatment.

## Conclusions

This case highlights the importance of recognizing inappropriate TSH secretion in patients who exhibit persistently elevated TSH levels despite high-dose thyroid hormone therapy. Greater awareness of the biochemical patterns and diagnostic pitfalls associated with thyrotroph PitNETs is essential to avoid misclassification and ensure timely management. In addition, this case provides a unique observation regarding the long-term stability and non-invasive behavior of a thyrotroph PitNET during prolonged exposure to supraphysiologic doses of levothyroxine. This unexpected tumor behavior raises the possibility that partial or heterogeneous sensitivity to thyroid hormone-mediated feedback may exist in a subset of these tumors, offering new insight into their biological variability.
